# Commissioning of a precision preclinical 200 kV x‐ray irradiator based on modular adaptations

**DOI:** 10.1002/mp.17758

**Published:** 2025-03-26

**Authors:** Lorenz Wolf, Peter Kuess, Sabine Leitner, Dietmar Georg, Barbara Knäusl

**Affiliations:** ^1^ Division of Medical Physics, Department of Radiation Oncology Medical University of Vienna Vienna Austria; ^2^ MedAustron Ion Beam Therapy Centre Wiener Neustadt Austria; ^3^ Competence Centre for Preclinical Imaging and Biomedical Engineering, Faculty of Health University of Applied Sciences Wiener Neustadt Austria; ^4^ Christian Doppler Laboratory for image and knowledge driven precision radiation oncology, Department of Radiation Oncology Medical University of Vienna Vienna Austria

**Keywords:** preclinical research, small animal irradiation, x‐ray

## Abstract

**Background:**

Preclinical research in radiation oncology encompasses a range of methodologies, including in vitro cell studies and in vivo small animal experiments, as well as in silico studies to evaluate radiation‐induced side effects and tumor responses.

**Purpose:**

This study addresses the need for high‐precision x‐ray irradiation solutions as reference for preclinical research. Modifications of an industrial kilovoltage x‐ray unit, along with the commissioning of a commercial treatment planning system (TPS), aimed to enable reliable irradiation of small animals in a horizontal beam geometry. All advancements enhancing the irradiation framework are made available, offering cost‐effective upgrades for existing systems.

**Methods:**

An industrial kilovoltage x‐ray unit was equipped with a dual collimation system, featuring a fixed primary and variable secondary collimators with aperture diameters of 5 to 30 mm. Additional modular adaptations were designed and manufactured, including a multimodal mouse bedding unit, a dedicated dosimetry phantom and a quality assurance (QA) phantom. Output factors, percentage depth dose curves and lateral dose profiles were acquired to generate a beam model in the TPSμ‐RayStation 8B (RaySearch Laboratories, Stockholm, Sweden), using a diamond detector and radiochromic films. Treatment plans for 10 mice were created, evaluated via dose‐volume metrics and the homogeneity index and subsequently dosimetrically compared to QA measurements through a gamma analysis with a 1%/1mm acceptance criterion.

**Results:**

The resulting beam model was validated within a maximum dose deviation of 1.7%. Aperture diameters close to potential target diameters were found to be effective for achieving sufficient target coverage in silico, as demonstrated for a 5 mm target with a homogeneity index of (9.9 ± 0.7)%. Dedicated QA measurements revealed a maximum dose deviation of 1.9% from the TPS and a median gamma passing rate of 100%, confirming the suitability of the proposed solution.

**Conclusions:**

Cost‐effective adaptations for an kilovoltage x‐ray irradiation framework were designed, manufactured and commissioned, and contribute to the accessibility of preclinical irradiation research. These components are integrated into a comprehensive preclinical particle beam platform.

## 1 INTRODUCTION

Preclinical research constitutes a pivotal element of radiation oncology and encompasses a diverse array of methods and techniques, ranging from in vitro experiments, involving various cell types,^1^ to animal models.^2^, ^3^

Many radiation‐induced side effects, for example, mucositis or urinary bladder dysfunction, are investigated in animal models by irradiating parts of the body with collimated x‐ray beams.^2^, ^3^, ^4^ Research with respect to tumor response poses even higher requirements on the precision and conformity of the irradiation modality.^5^ In recent years, there has been a growing interest in the field of preclinical particle therapy.^6^, ^7^ Preclinical studies with charged particles are motivated by a variety of open radiobiological questions, such as the prominent example of relative biological effectiveness (RBE), which describes the ratio of absorbed doses that produce the same biological effect between a reference (typically megavoltage photons) and particle irradiation.^8^, ^9^, ^10^

Radiobiological experiments comparing multiple irradiation modalities necessitate reference irradiation in an identical experimental setting. Due to the small size and shallow position of targets in the preclinical context, the reference irradiation needs to be translated from megavoltage linear accelerators (LINACs) to dedicated kilovoltage x‐ray units.^11^ This growing demand of high‐precision x‐ray units triggered the evolution of dedicated kilovoltage x‐ray irradiators.^12^, ^13^ Additional efforts to directly dock these systems to existing ion beam infrastructure have been reported.^14^, ^15^, ^16^ Moreover, dedicated treatment planning systems (TPS) became recently available, integrating computed tomography (CT) images with a sub‐millimeter resolution.^17^ Commissioning of such dedicated preclinical kilovoltage x‐ray units poses additional dosimetric challenges, as the recommendations from international guidelines cannot always be met due to deviating geometric dimensions and treatment volumes. This is of particular importance in the frame of RBE studies, as dosimetric errors in the reference field directly translate to the RBE.^18^, ^19^

In addition to a reference kilovoltage x‐ray irradiation unit, state‐of‐the‐art preclinical in vivo research often necessitates access to preclinical imaging modalities. Furthermore, in vivo experiments require on‐site animal housing or high‐end biosecurity measures to enable the irradiation of animals from an external animal facility. Such a fully‐equipped multidisciplinary research facility is scarce, prompting researchers to adapt and leverage the available equipment to its fullest potential.

In this study, we demonstrate the modification of an industrial kilovoltage x‐ray unit, initially not intended for preclinical research, to replicate the setup of a horizontal particle beam line for reference irradiations, including a beam collimator for various field sizes. Mimicking the same irradiation setup allows to perform preclinical experiments with identical beam incidence angles but using different beam qualities. By doing so, a more systematic comparison of the biological experiments, positioning of the underlying model and thus the resulting biological effect is given. Basic beam data for a beam model in a dedicated TPS were acquired using a down‐scaled water‐equivalent dosimetric slab phantom. For in vivo studies, a modular, low‐cost, 3D‐printed mouse bedding unit was developed and complemented by a tissue‐equivalent QA phantom for streamlined treatment verification. Finally, the entire framework was tested in a pilot treatment planning study with subsequent subject specific QA measurements.

## 2 MATERIAL AND METHODS

### 2.1 X‐ray irradiator and beam collimation

An oil‐cooled YXLON Maxishot (YXLON International GmbH, Hamburg, Germany) full‐protection kilovoltage x‐ray unit, able to deliver 10 to 200 kV beams, was used in this study.^20^ The x‐ray tube has been mounted to the side of the irradiation unit to mimic the experimental setting of a horizontal particle therapy beam line. The tungsten target angle was 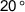 with a focal spot size of 5.5mm, while filtration consisted of 3mm Be, 3mm Al, and 0.5mm Cu. The diameter of the beam exit window was 120mm. All measurements were performed with 200kV and 20mA—settings suitable for the irradiation of cells and small animals.^2^, ^4^ The dose delivery was controlled via a timer that can be adjusted in 1s increments.

**FIGURE A1:**
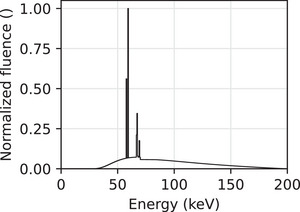
200kV x‐ray spectrum calculated with SpekPy (version 2.0.11.1) using a Tungsten target with an anode angle of 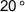 and 3mm Be, 3mm Al, and 0.5mm Cu filtration.

The spectrum of the x‐ray unit was generated using the Python library SpekPy (v2.0.11.1),^21^, ^22^, ^23^ using the nominal values for tube voltage, target angle and filtration, yielding half‐value layer (HVLs) of 1.13mm(Cu) and 12.56mm(Al) (Figure A1).

**FIGURE 1:**
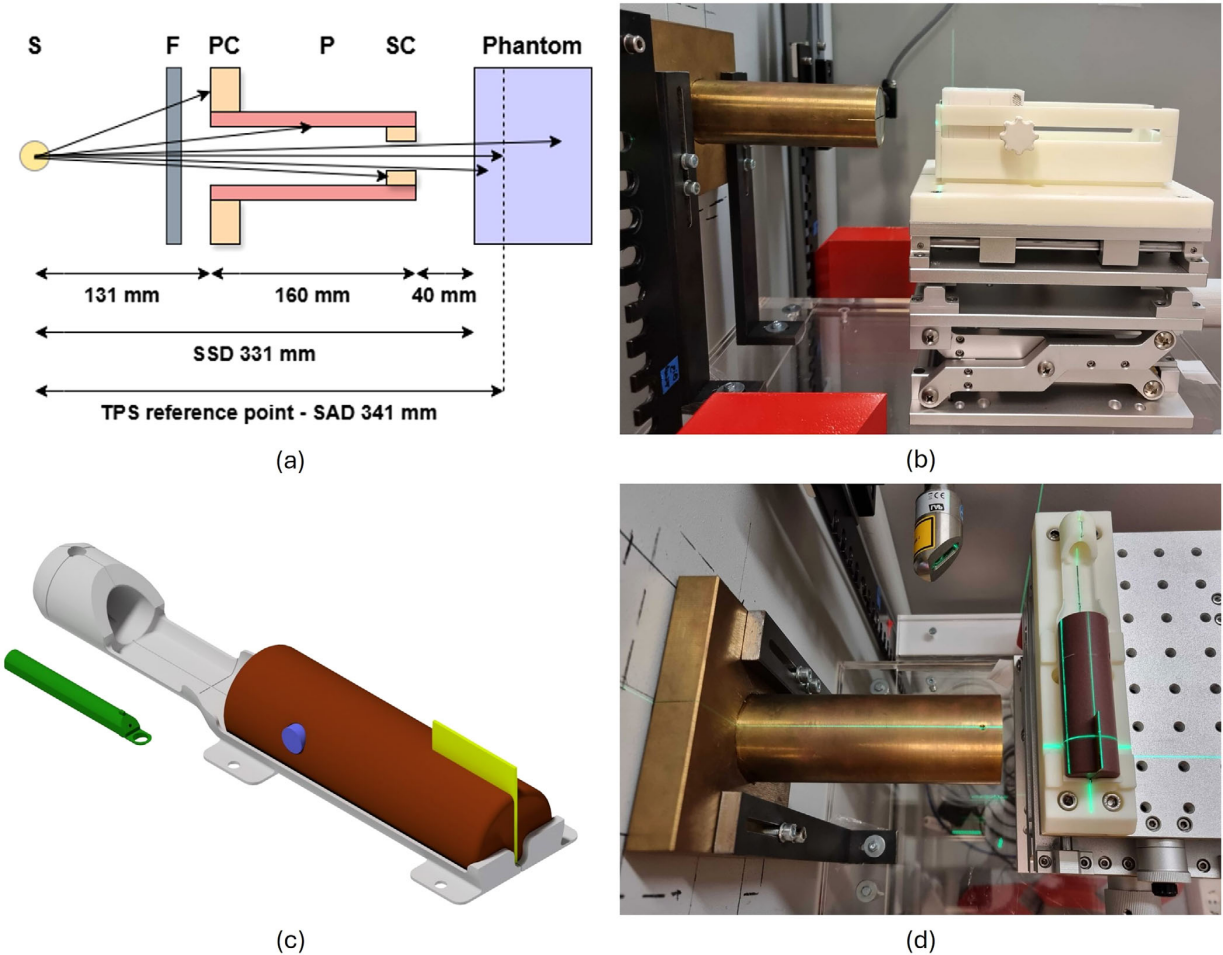
(a) Schematic of the collimation geometry indicating the source (S), filter (F), PC, pipe (P), SC, and a phantom. The TPS reference point was defined 50mm downstream of the SC, in a depth of 10mm. (b) Collimator and SFDP were mounted on the positioning stage. The phantom could be interchanged with the mouse bedding unit. (c) Schematic of the QA phantom (brown) mounted to the mouse bedding unit (grey). A detector (blue) or radiochromic films (yellow) can be inserted in the phantom for QA measurements of lateral beams. The tooth bar, which can be fixed in the nose cone to allow longitudinal fixation of the animals, is shown in green. (d) QA phantom (including inserted EBT3 films) mounted on the mouse bedding unit. PC, primary collimator; QA, quality assurance; SC, secondary collimator; SFDP, small field dosimetry phantom; TPS, treatment planning system.

For precise positioning within the unit, a vertical cross laser and a horizontal line laser (LAP GmbH Laser Applikationen, Lüneburg, Germany) have been installed inside the irradiation cabinet. To achieve variable collimation of the beam, a brass dual collimation system was implemented, comprising a primary collimator (PC) with 46mm aperture diameter and interchangeable secondary collimators (SCs) (Figure 1a). A 160mm long brass tube interconnected the PC and SC, providing a mounting mechanism for the latter. Multiple SCs with a thickness of 20mm were manufactured, covering typical aperture diameters employed in preclinical research: 5, 7, 8, 10, 15, 20, 25, and 30 mm. Computer‐aided design (CAD) files for the collimation system are available online (https://doi.org/10.5281/zenodo.12805247).

### 2.2 Irradiation setup and QA phantoms

Modular devices were developed to enable a straightforward irradiation of small animals and dosimetry in the presented kilovoltage x‐ray unit. A high‐precision linear stage (Toplionace, Shenzhen, China) was used to align the equipment downstream of the collimator using dedicated adapter plates.

A bedding unit for mice (Figure 1c) was developed to ensure accurate and reproducible animal positioning for high‐precision in vivo studies. This bedding unit was designed to be compatible with all steps in a typical preclinical workflow, including CT, Magnetic Resonance (MR), Positron emission tomography (PET) imaging and irradiation. Key features included a nose cone and a hollow tooth bar. The tooth bar could be introduced into the nose cone to fix the animals' jaws, ensuring consistent longitudinal positioning while offering the possibility of introducing gaseous anaesthesia. The maximum diameter of the nose cone was 22.5mm and thus compatible with the small bore sizes of preclinical imaging devices.

A small field dosimetry phantom (SFDP) was designed as a down‐scaled, water‐equivalent slab phantom (Figure 1b). RW3 slabs (PTW‐Freiburg, Freiburg, Germany) with dimensions of 60 × 60 ${\mathrm{mm}}^{2}$ and thicknesses of 1, 5 *and* 10 mm, summing up to a maximum thickness of 160 mm, could be mounted within the phantom frame. Dedicated adapters for various detectors were manufactured to ensure precise alignment, as well as a consistent amount of backscattering material of 20 mm thickness, which resembles a typical preclinical scenario. Radiochromic films could be placed between the slabs at the desired depth. Lateral and longitudinal markers were included for laser alignment.

The SFDP frame and bedding unit were 3D‐printed from acrylonitrile butadiene styrene (ABS) using the fused deposition modeling (FDM) technique on a Bambu Lab X1‐Carbon (Bambu Lab EU GmbH, Frankfurt am Main, Germany) printer. ABS (Sunlu International Hong Kong Holdings Ltd., Hong Kong, China) was used due to its favorable mechanical properties and adequate dosimetric properties in x‐ray as well as proton beams.^24^, ^25^

To verify the geometric and dosimetric accuracy prior to any in vivo experiments, a QA phantom was established (Figure 1c,d). The design vaguely mimicked the shape and dimensions of a mouse, while still allowing for straightforward positioning on the bedding unit and thereby for an efficient QA workflow. The maximum dimensions were 93 × 29 × 21 mm^3^. The phantom featured inserts for a detector of 7mm diameter in axial orientation, as well as a stack of three radiochromic films ($30\times 30{\mathrm{mm}}^{2}$) in orthogonal orientation. Assuming lateral irradiation and a detector entrance window of 1mm, the films and sensitive volume of the detector were located at a maximum depth of 14.4mm. The entire phantom was manufactured from a solid piece of water‐equivalent material (Solid Water HE; Sun Nuclear Corporation, Melbourne, Florida, USA). CAD files for the mouse bedding unit, SFDP and QA phantom are provided online (https://doi.org/10.5281/zenodo.12805247).

### 2.3 Dosimetry and basic beam data

A type 30013 Farmer ionization chamber (PTW‐Freiburg, Freiburg, Germany) was calibrated at a secondary standard laboratory in an x‐ray beam at 180kV (HVL = 1mm Cu) and 250kV (HVL = 2.6mm Cu) in terms of air kerma free in air, resulting in an identical calibration coefficient for both beam qualities. Thus, this coefficient could be used to cross‐calibrate this chamber to a PTW type 60019 microDiamond detector (mD) and a type 34045 Advanced Markus Chamber in 200kV (HVL = 1.13mm Cu) according to IAEA TRS‐398 recommendations.^19^ However, not all recommendation could be fulfilled due to geometrical constraints within our device. In particular, a field size of 20cm diameter and a source‐to‐surface distance (SSD) of 331mm were used. The air kerma calibration was converted to dose to water using the mean water to air ratios of the mass energy absorption coefficients provided by Andreo et al., as well as the chamber perturbation factors provided by Bancheri et al., as outlined in the TRS‐398.^19^, ^26^, ^27^

Percentage depth dose curves (PDDs) were measured using the mD, which was shown to be suitable for small kilovoltage x‐ray beams.^28^ For this purpose the detector was mounted in the SFDP, which was positioned 40mm downstream of the SC, corresponding to a SSD of 331mm (Figure 1a). PDDs were acquired for all available SCs in three repetitions (without altering the setup), for depths ranging from 1 to 50 mm in steps of 5mm. Output factors were measured in a depth of 20mm RW3 to replicate the reference conditions according to the IAEA TRS‐398 protocol as close as possible. Still, the preclinical setting necessitated a reference field size of 42mm for output factor measurements, as well as usage of the non‐cylindrical mD detector, deviating from the recommended configuration.^19^ Dose rates for the 8mm reference aperture were measured in a depth of 10mm of RW3 at the TPS reference point (see Section 2.4) for timer settings ranging from 5 to 330 s, corresponding to accumulated doses of approximately 0.1 to 10 Gy.

To assess dose variations peripheral to the central beam axis, lateral dose profile (LDP) measurements were acquired for all apertures and six depths using Gafchromic EBT3 films (Ashland, Wayne, NJ, USA). The film batch (lot #3082203) was calibrated against the Advanced Marcus chamber for 200kV at dose levels of 0.5, 1.0, 1.5, 3.0, 5.0, 7.0, and 10.0 Gy. An Epson Expression 11000XL flatbed scanner (Seiko Epson Corporation, Nagano, Japan) was employed for image acquisition. Scanning procedures were conducted prior to and 24h after irradiation with a resolution of 300dpi. Film handling procedures were executed following the guidelines described in the AAPM TG 235 report^29^ and in previous studies,^30^, ^31^ achieving standard practices. Horizontal and vertical profiles were extracted from the films by averaging three individual line profiles centered symmetrically around the distributions' center, which were subsequently smoothed via a median filter (three pixels window) to reduce the influence of film noise. The resulting profiles were analyzed in terms of field size (full width at half maximum (FWHM)), penumbra, and flatness.^32^ The penumbra was calculated as the distance between the points of 80% and 20% of maximum dose. Flatness (F) was calculated as the percentage difference between the maximum ($D_{\max}$) and minimum ($D_{\min}$) values across the profile within the inner 80% of the FWHM as

(1) $$F=\frac{D_{\max}-D_{\min}}{D_{\max}+D_{\min}}\times 100.$$

### 2.4 Beam model

The beam model was created and validated for µ‐RayStation 8B (RaySearch Laboratories, Stockholm, Sweden), which is dedicated to the irradiation of small animals and included the following features: GPU based Monte Carlo (MC) dose engine for kilovoltage beams (v1.0, dose to water, statistical uncertainty of 0.5%), dose grid resolution of 0.2mm, structure and aperture resolution of 0.1mm.^17^ Necessary beam model inputs for the TPS comprised the x‐ray spectrum (Section 2.1), as well as PDDs and relative LDPs for all available SCs (Section 2.3). Absolute dose calibration was defined via output factors relative to the 8mm aperture and a steady dose rate measured at the TPS reference point, defined 50mm downstream of the SC in a depth of 10mm, at a resulting source‐to‐axis distance (SAD) of 341mm (Figure 1a).

For beam model validation, treatment plans were created on a virtual RW3 phantom for the three major apertures of 5, 8 *and* 30 mm diameter. All plans were scaled to a dose of 1Gy at the TPS reference point and the predicted irradiation time served as a basis for validation measurements in depths of 1, 5, 10, 20 *and* 50 mm using the mD. The relative dose agreement of simulation to measurement was investigated and one‐way analysis of variance (ANOVA) was performed to identify potentially significant differences for the various depths and apertures. Test statistics were computed using the SciPy library (version 1.11.3) in Python, with significance determined for *p*‐values less than 0.05.^33^

### 2.5 Subject specific treatment planning and dosimetric verification

The translation of the dosimetric characteristics into the in vivo context was investigated for 10 BALB/cJRj mice (Ethics approval from the Federal Ministry of Education, Science and Research of Austria: 2023‐0.556.474). All animals were placed on the mouse bedding unit and received a spiral cone beam μCT scan (X‐CUBE; Molecubes, Gent, Belgium) using a 50kV tube voltage and 0.225mA current at a resolution of $0.2\times 0.2\times 0.2{\mathrm{mm}}^{3}$ for treatment planning. For this purpose, a Hounsfield units (HU) calibration curve was established based on tissue‐equivalent materials (CIRS 062M; Sun Nuclear Corporation, Melbourne, Florida, USA).^25^ Dose calculation settings were equivalent to those described in Section 2.4

A horizontal beam employing a 5mm aperture was delivered from the right side to emulate partial brain irradiation with the isocenter and dose normalization point aligned with the center of the right hippocampus. The dose prescription to the normalization point was 1.0Gy. Target coverage was assessed with three cylindrical structures (diameters of 4, 5 *and* 6 mm), which were centered around the normalization point and intersected with the right hemisphere of the brain. To evaluate dose in the surrounding tissue, the two brain hemispheres and the skull (threshold based 250 to maximum HU) were delineated.

Dose profiles in beam direction through the normalization point were analyzed for each treatment plan and subsequently averaged for all 10 rodents. $D_{10\%}$ and $D_{90\%}$ were determined additionally as surrogates for the maximum and minimum dose in the small target volumes (< $0.11\;\;cm^{3}$). Together with $D_{50\%}$ the homogeneity index (HI) was calculated as a metric for target coverage.^34^

(2) $$HI=\frac{D_{\text{10}\%}-D_{\text{90}\%}}{D_{\text{50}\%}}$$

The generated treatment plans were re‐calculated on a mimicked μCT of the QA phantom (virtual air μCT complemented with the structures of the phantom) (Section 2.2). Each plan was subsequently delivered twice: once targeting the mD and once a stack of three EBT3 films placed within the phantom. The dose measured by the detector was compared to the prediction of the TPS in terms of relative dose deviation. The 2D dose distribution of the films was resampled to the μCT resolution and aligned to the corresponding delineated μCT structure utilizing the Python interface of the SimpleITK toolkit (version 2.3.1).^35^, ^36^ A 2D gamma analysis was then conducted to compare the doses of the TPS and film, employing the PyMedPhys library (version 0.38.0) with a 1% dose difference (DD) and 1mm distance to agreement (DTA) acceptance criterion at a lower dose cutoff of 10%.^37^

## 3 RESULTS

### 3.1 X‐ray beam characteristics

**FIGURE 2:**
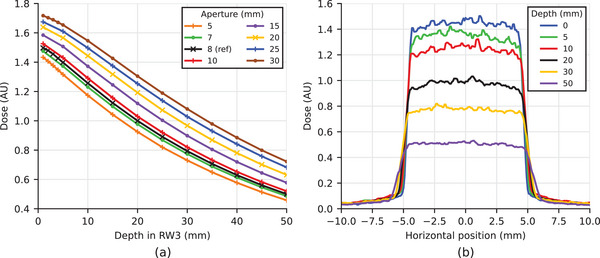
(a) PDD acquired with the mD and normalized to the dose measured in 20mm depth using the reference aperture (8 mm). Each marker corresponds to three repeated measurements—the respective relative standard deviations were below 0.2%. (b) LDPs for the reference aperture measured with EBT3 films were normalized to the dose measured with the mD on the central axis in 20mm depth. mD, microDiamond detector; PDD, percentage depth dose curve; LDPs, lateral dose profiles.

The acquired PDDs, normalized to the dose measured with the reference aperture (8mm) in a depth of 20mm, are shown in Figure 2a. Relative differences comparing the largest to smallest aperture increased from 17% at 1mm to 37% at 50mm depth. The distance from the surface to the 95% dose fall‐off was 3.5mm for the 5mm aperture and 6mm for the 30mm aperture. The corresponding output factors ranged from 0.92 to 1.30 as listed in Table 1. Figure 2b shows LDPs for the reference aperture.

**TABLE 1:** Beam parameters in a depth of 20mm in RW3.

Aperture (mm)	Output factor	FWHM (mm)	Penumbra (mm)	Flatness (%)
5	0.92	6.1	0.6	3.6
7	0.98	8.7	0.6	4.5
8	1.00	10.0	0.7	5.0
10	1.03	12.4	0.6	4.6
15	1.12	18.6	0.8	6.0
20	1.19	24.8	0.9	6.3
25	1.25	31.4	1.2	5.9
30	1.30	37.3	1.3	7.3

*Note*: Output factors were normalized to the reference field employing the 8mm aperture.

Abbreviation: FWHM, full width at half maximum.

Field size, penumbra, and flatness for all apertures are shown in Table 1 for a depth of 20mm. The ratio of aperture to field size was (1.24 ± 0.01), thus varying by less than 1% for all diameters. The sharpest penumbra was observed at the surface and increased with depth by up to 2% and 5% for the smallest and largest aperture, respectively.

Measurements for 10 timer settings of 30 to 330 s yielded a mean dose rate of ($1.722\pm 0.002)\mathrm{Gy}\;\min^{-1}$, showing a constant dose rate at a depth of 10mm for a total dose between 0.9 and 10 Gy. Increased dose rates were found for irradiation times smaller than 30s, with deviations of up to 4% for 5s. Accordingly, each 1s increment on the x‐ray unit's timer corresponds to an approximate dose increment of 0.03Gy.

A dosimetric uncertainty budget was established in accordance with the recommendations of the TRS‐398 protocol for medium energy x‐rays,^19^ which is shown in Table 2. Daily output variations of the x‐ray unit were measured and accounted for with respective correction factors and are therefore excluded from the budget.

**TABLE 2:** Dosimetric uncertainty budget for beam model data acquisition.

Source	Type	Value (%)
Reference chamber calibration^a^	B	1.4
Mass energy absorption coefficient (water to air ratio)^b^	B	0.5
Chamber perturbation factor^c^	B	1.5
Beam quality factor^a^	B	0.5
Detector cross‐calibration^d^	A	0.2
Beam data acquisition repeatability^d^	A	0.2
Temperature dependence of microDiamond^e^	B	0.1
Establishment of reference conditions^f^	B	1.0
Backscattering conditions (dependent of field size)^g^	B	0.5–4.0
**Total 1‐** σ **standard uncertainty**	**Combined**	**2.4–4.7**

*Note*: To account for deviating backscattering conditions during basic beam data acquisition and detector cross‐calibration, an additional uncertainty was included. The contribution of the backscattering conditions depends strongly on the field size, ranging from 0.5% for field diameters ≤ 10mm up to 4% for diameters of 42mm. Thus the total uncertainty is given as a range for small to large field sizes.

^a^ Provided by secondary standard laboratory.

^b^ According to TRS‐398 and Andreo et al.^19^, ^26^

^c^ According to TRS‐398 and Bancheri et al., including field size dependence.^19^, ^27^

^d^ Measured quantity.

^e^ Specified as $0.05\;\%{/}^{\circ }C$, assuming irradiation cabinet temperature within ± 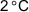.

^f^ According to TRS‐398, accounting for dose gradients up to 1%/mm.

^g^ Interpolated from tabulated values provided by Chen et al. for field diameters ≤ 42mm.^38^

### 3.2 Beam model validation

Validation for aperture diameters of 5, 8 *and* 30 mm at depths of 1, 5, 10, 20, and 50 mm showed relative dose deviations within −1.5% to 1.7% comparing measurements and dose calculation, indicating good agreement for all apertures and depths within the dosimetric uncertainties. A one‐way ANOVA showed no significant differences in dose agreement for the three validated apertures (p=0.9). However, ANOVA revealed differences in mean dose agreement for the investigated depths (p=0.0005), with a trend shifting from dose underestimation at 1mm to overestimation at 50mm.

### 3.3 In silico dose calculation for mice

**FIGURE 3:**
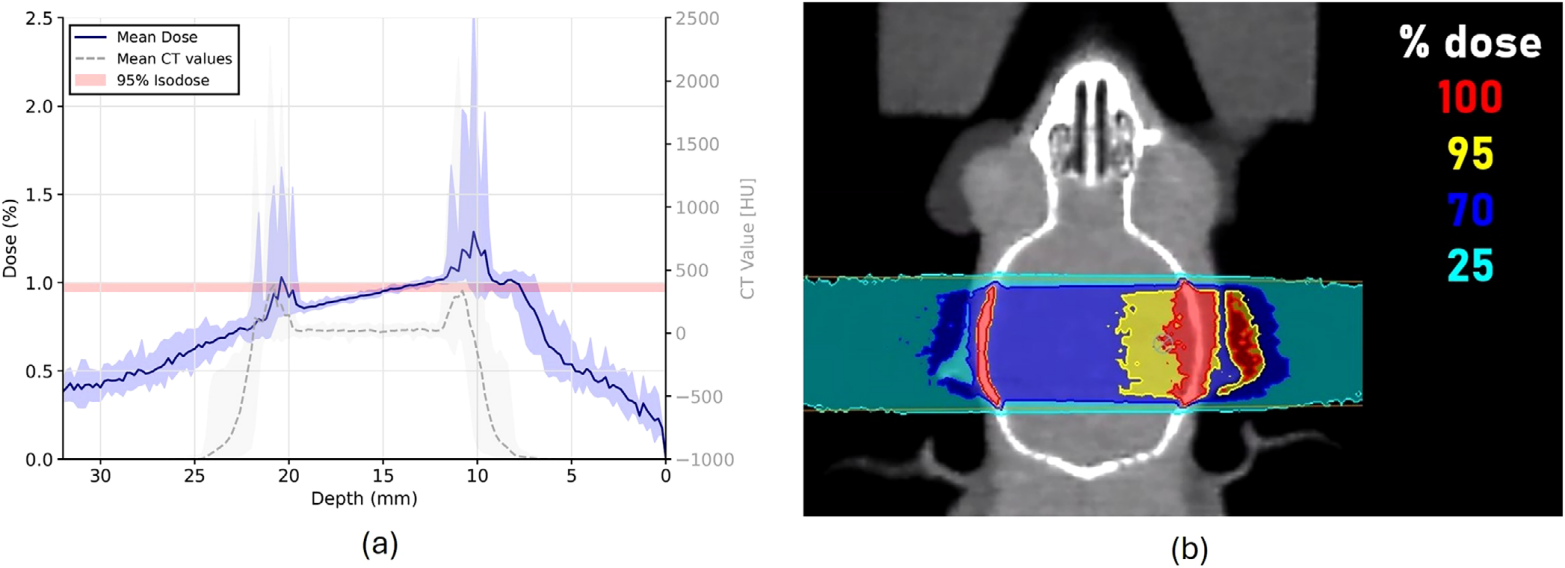
(a) Average line profiles of dose and HU in beam direction for all mice. The confidence intervals indicate the minimum and maximum values. The peaks of the dose and HU profiles in an approximate depth of 11 and 21 mm depict the high dose deposition in the skull. Note that the horizontal axis was flipped to emphasize the beam incident direction from the right side. (b) Coronal view of a dose distribution overlayed on a μCT of an exemplary mouse. HU, Hounsfield unit.

**FIGURE A2:**
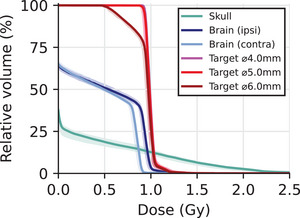
Averaged dose‐volume histogram for 10 mice with confidence intervals indicating the minimum and maximum values.

The treatment plans for 10 mice showed very similar dose distributions, which was reflected in a low relative standard deviation of the near minimum and maximum dose below 1.2% for the target and the two brain hemispheres. The narrow confidence intervals of the averaged dose‐volume histogram (DVH) curves indicate a high anatomical similarity between the mice (Figure A2). The averaged dose profile in beam incident direction in Figure 3a reveals the highest dose depositions in the skull, with maximum values of up to 2.93Gy for one individual (prescribed dose of 1Gy). An exemplary dose distribution in coronal view is shown in Figure 3b.

The 95% isodose reached a depth of 5mm in the ipsilateral brain hemisphere falling to 85% in the distal part of the contralateral side. The average FWHM of the lateral profile through the isocenter was (5.97 ± 0.02) mm and was thereby in agreement with the FWHM of the commissioning measurements in RW3 as reported in section 3.1. Good target coverage was reflected by the comparable HI of (9.2 ± 0.3)% and (9.9 ± 0.7)% for the targets with 4 and 5 mm diameter. For a diameter of 6 mm the HI increased to (30.5 ± 0.8)%.

### 3.4 Quality assurance

Plan specific QA measurements with the mD revealed a dose underestimation of the TPS for all 10 treatment plans with a mean dose deviation of (−1.74 ± 0.04)% and a maximum absolute deviation of 1.90%. With three films per plan, a total of 30 films were irradiated, resulting in a median gamma passing rate of 100% using a passing criterion of 1%/1 mm. The lowest passing rate for an individual plan was (96 ± 3)%, while for five plans the passing rate of all three films was 100%.

## 4 DISCUSSION

A beam collimation system for a kilovoltage x‐ray unit with a horizontal beam was designed and manufactured and complemented by a mouse bedding unit and respective dosimetric and QA phantoms. These components are integrated as part of a preclinical platform for ion beam reference irradiation. The mouse bedding ensured reproducible animal positioning throughout the entire workflow, ranging from multimodal imaging to irradiation. The designed x‐ray collimators' primary purpose was to mimic the available particle beam irradiation geometry and by that to enable a direct comparison of the biological effect when using different beam qualities in biological experiments.^39^ Thus, a purely horizontal x‐ray beam line for reference irradiation was intended.

Even though the presented system is less sophisticated compared to commercial preclinical systems, we obtained comparable dosimetric characteristics. Lindsay et al., described results for a 225kVp unit (X‐RAD 225Cx, Precision X‐Ray Inc., USA), reporting penumbrae of 0.6 to 0.7 mm for apertures of 10mm diameter and 0.5 to 0.7 mm for aperture diameters of 5mm in a depth of 5mm, which is in good agreement with the results presented in this study. Their study showed lower values for profile flatness, however, they also discussed the limited comparability of the flatness parameter due to its sensitivity to film processing protocols and analysis.^40^

The dose rate of our system was (1.722 ± 0.002) Gy $\min^{-1}$ and is thus comparable to multiple established solutions, where dose rates between 1 and 4 Gy $\min^{-1}$ have been reported.^13^, ^41^, ^42^, ^43^ Increases in dose rate of up to 4% were identified for irradiation times smaller than 30s. Consequently, this should be taken into account for dose levels below 0.9Gy.

The spectrum calculated via SpekPy was used as input for the TPS. Considering tolerances of the nominal parameters (as specified by the vendor) of the used x‐ray unit (2kV high voltage, 0.005mm filter thickness, and 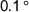 target angle), we estimated an uncertainty of 2% resulting in a calculated HVL of (1.13 ± 0.02) mm Cu. Respective measurements revealed a HVL of (1.09 ± 0.03) mm Cu. Although HVL calculation and measurement agree within the respective uncertainties, offsets in HVL, caused by deviations of the calculated compared to the actual spectrum, could result in inaccurate dose calculation.

Beam model validation showed good absolute agreement between dose calculation and measurement within 1.7%, demonstrating the suitability of the established model. Notably, dose underestimation close to the phantom surface and overestimation at depths up to 50mm was identified and was most likely caused by imperfect modeling of the x‐ray spectrum and measured beam profiles.

While the IAEA Code of Practice TRS‐398 guidelines for kilovoltage x‐ray beam dosimetry are based on the absorbed dose to water formalism, standard calibration laboratories that can provide these standards are still scarce and thus absorbed dose determination for kilovoltage x‐rays commonly still relies on air kerma calibrations, as performed in the presented study. The air kerma based measurements were converted to absorbed dose to water, as outlined in Section 2.3, this procedure introduced additional uncertainties.

The established dosimetric uncertainty budget comprises factors from the detector calibration chain, temperature dependencies of the detector response, establishment of reference conditions as well as deviating backscattering conditions and was based on the TRS‐398 recommendations. The thickness of material behind the detector to provide full backscatter is roughly equal to the field size. The in‐house developed dedicated adapter provides 20mm of backscattering material. Thus, full backscatter conditions could be assumed for the smallest field sizes ≤ 10mm, but not for field sizes ≥ 20mm. The tabulated values of field size dependent backscatter factors provided by Chen et al. allowed us to introduce an uncertainty component accounting for this fact, resulting in an estimated maximum 1‐σ standard uncertainty of 4.7%.^19^, ^38^

Basic beam data, such as PDDs and output factors were determined using the mD. Its suitability for kilovoltage x‐ray beam dosimetry was shown by Damodar et al. However, Kaveckyte et al. also pointed out that synthetic diamond detectors show a non‐negligible variation in the relative intrinsic energy response for low energy photons.^28^, ^44^ The additional beam hardening filtration of the used x‐ray unit minimizes the contribution of these low energy photons to the total spectrum (Figure A1) and thus justifies usage of the mD for dosimetry in the outlined setting.

The sharp penumbra of 0.6mm for the smallest fields indicated that apertures with diameters close to potential target diameters were appropriate to achieve sufficient lateral target coverage and thus support sparing of surrounding tissues. The irradiation plans for the in silico mouse brain study were consequently generated using a 5mm SC. This beam collimation enabled good coverage of the target region, reflected in a HI of (9.9 ± 0.7)% for a 5 mm target diameter. A high anatomical similarity of the animals was reflected in the comparable dose parameters and is the ideal basis for a standardized workflow tailored to a dedicated research question.

Different aperture shapes (e.g. rectangular) might prove beneficial for healthy tissue sparing for particular anatomical sites and will be subject of future investigations. For potential target sizes below diameters of 5mm, the commissioning data needs to be complemented. However, the suitability of the mD needs to be investigated for these field sizes. Alternatively, PDD and output factor measurements could be obtained with EBT3 films, thermoluminescent dosimeters or small volume scintillator detectors.

The developed QA phantom was designed to be compatible with the mouse bedding unit, allowing for a streamlined QA workflow in multiple beam modalities and eliminating the need for significant setup alterations between treatment verification and in vivo irradiation. Our QA results indicated that the outlined framework is well‐suited for in vivo experiments employing aperture diameters down to 5 mm. While the suggested phantom was aimed at practicability, more advanced, anatomical mouse phantoms have been reported previously and can be employed to additionally investigate the influence of tissue heterogeneities.^45^, ^46^

Currently, the proposed framework supports a landmark‐based positioning approach using isocentric lasers. A regular QA procedure to confirm the laser alignment with the beam isocenter has not been introduced so far. To overcome this limitation radiochromic films were used in combination with a cross‐hair collimator to validate the laser position in this study. For target diameters less than 5 mm, sub‐millimetric positioning accuracy, as provided by modern commercial solutions, is crucial.^47^, ^48^ However, achieving this accuracy will be challenging without online image‐guidance capabilities, which is the main limitation of the presented solution. Future studies will explore individual restraining jigs and potentially 3D printed body molds to address this limitation.^49^

The introduced modular adaptations can be manufactured and adapted by other users of similar systems, allowing for fast implementation and enhancements within the preclinical research community.

## 5 CONCLUSION

We proposed enhancements for an industrial kilovoltage x‐ray unit to enable high‐precision irradiation of small animals with a commercial TPS for dose calculation. This upgraded system includes a collimation system with interchangeable apertures, a mouse bedding unit, a dosimetric and a QA phantom. These modular designs, combined with the presented workflow and its validation, as well as the in silico study provide a cost‐effective and accessible contribution to preclinical ion beam platforms aiming for improved reproducibility, comparability, and consistency within this research field.

## CONFLICT OF INTEREST STATEMENT

Dietmar Georg serves as a deputy editor for the journal *Medical Physics*. The Department of Radiation Oncology (Medical University of Vienna) has an institutional research contract with RaySearch Laboratories (Stockholm, Sweden).

## References

[1] Mara E , Clausen M , Khachonkham S , et al. Investigating the impact of alpha/beta and LET on relative biological effectiveness in scanned proton beams: an in vitro study based on human cell lines. Med Phys. 2020;47:3691‐3702. 32347564 10.1002/mp.14212 PMC7496287

[2] Frings K , Gruber S , Kuess P , Kleiter M , Dörr W . Modulation of radiation‐induced oral mucositis by thalidomide. Strahlenther Onkol. 2016;192:561‐568. 27282278 10.1007/s00066-016-0989-5

[3] Gruber S , Frings K , Kuess P , Dörr W . Protective effects of systemic dermatan sulfate treatment in a preclinical model of radiation‐induced oral mucositis. Strahlenther Onkol. 2018;194:675‐685. 29497792 10.1007/s00066-018-1280-8 PMC6008363

[4] Kowaliuk J , Sarsarshahi S , Hlawatsch J , et al. Translational aspects of nuclear factor‐kappa B and its modulation by thalidomide on early and late radiation sequelae in urinary bladder dysfunction. Int J Radiat Oncol Biol Phys. 2020;107:377‐385. 32035188 10.1016/j.ijrobp.2020.01.028

[5] Verhaegen F , Butterworth KT , Chalmers AJ , et al. Roadmap for precision preclinical x‐ray radiation studies. Phys Med Biol. 2023;68:06RM01. 10.1088/1361-6560/acaf45 36584393

[6] Dosanjh M , Jones B , Pawelke J , Pruschy M , Sørensen BS . Overview of research and therapy facilities for radiobiological experimental work in particle therapy. Report from the European Particle Therapy Network radiobiology group. Radiother Oncol. 2018;128:14‐18. 29703500 10.1016/j.radonc.2018.03.008

[7] Parodi K , Assmann W , Belka C , et al. Towards a novel small animal proton irradiation platform: the SIRMIO project. Acta Oncol. 2019;58:1470‐1475. 31271091 10.1080/0284186X.2019.1630752

[8] Paganetti H . Mechanisms and review of clinical evidence of variations in relative biological effectiveness in proton therapy. Int J Radiat Oncol Biol Phys. 2022;112:222‐236. 34407443 10.1016/j.ijrobp.2021.08.015 PMC8688199

[9] Ilicic K , Combs S , Schmid T . New insights in the relative radiobiological effectiveness of proton irradiation. Radiat Oncol. 2018;13:6. 29338744 10.1186/s13014-018-0954-9 PMC5771069

[10] Mohamad O , Sishc BJ , Saha J , et al. Carbon ion radiotherapy: a review of clinical experiences and preclinical research, with an emphasis on DNA damage/repair. Cancers. 2017;9:66. 28598362 10.3390/cancers9060066 PMC5483885

[11] Koontz BF , Verhaegen F , Ruysscher DD . Tumour and normal tissue radiobiology in mouse models: how close are mice to mini‐humans? Br J Radiol. 2017;90:20160441. 27612010 10.1259/bjr.20160441 PMC5605019

[12] Tillner F , Thute P , Bütof R , Krause M , Enghardt W . Pre‐clinical research in small animals using radiotherapy technology—a bidirectional translational approach. Z Med Phys. 2014;24:335‐351. 25125191 10.1016/j.zemedi.2014.07.004

[13] Verhaegen F , Granton P , Tryggestad E . Small animal radiotherapy research platforms. Phys Med Biol. 2011;56:R55‐R83. 21617291 10.1088/0031-9155/56/12/R01

[14] Ford E , Emery R , Huff D , et al. An image‐guided precision proton radiation platform for preclinical in vivo research. Phys Med Biol. 2016;62:43. 27973343 10.1088/1361-6560/62/1/43

[15] Kim MM , Irmen P , Shoniyozov K , et al. Design and commissioning of an image‐guided small animal radiation platform and quality assurance protocol for integrated proton and x‐ray radiobiology research. Phys Med Biol. 2019;64:135013. 31075786 10.1088/1361-6560/ab20d9 PMC8690893

[16] Schneider M , Schilz JD , Schürer M , et al. SAPPHIRE—establishment of small animal proton and photon image‐guided radiation experiments. Phys Med Biol. 2024;69:095020. 10.1088/1361-6560/ad3887 38537301

[17] Chiavassa S , Nilsson R , Clément‐Colmou K , Potiron V , Delpon G , Traneus E . Validation of the analytical irradiator model and Monte Carlo dose engine in the small animal irradiation treatment planning system μ‐RayStation 8B. Phys Med Biol. 2020;65:035006. 31829982 10.1088/1361-6560/ab6155

[18] Subiel A , Patallo IS , Palmans H , et al. The influence of lack of reference conditions on dosimetry in pre‐clinical radiotherapy with medium energy x‐ray beams. Phys Med Biol. 2020;65:085016. 32109893 10.1088/1361-6560/ab7b30

[19] International Atomic Energy Agency . Absorbed Dose Determination in External Beam Radiotherapy, Technical Reports Series No. 398 (Rev. 1), IAEA, Vienna; 2024. 10.61092/iaea.ve7q-y94k

[20] Kuess P , Bozsaky E , Hopfgartner J , Seifritz G , Dörr W , Georg D . Dosimetric challenges of small animal irradiation with a commercial x‐ray unit. Z Med Phys. 2014;24:363‐372. 25270978 10.1016/j.zemedi.2014.08.005

[21] Bujila R , Omar A , Poludniowski G . A validation of SpekPy: a software toolkit for modelling x‐ray tube spectra. Phys Med. 2020;75:44‐54. 32512239 10.1016/j.ejmp.2020.04.026

[22] Poludniowski G , Omar A , Bujila R , Andreo P . Technical note: SpekPy v2.0‐a software toolkit for modeling x‐ray tube spectra. Med Phys. 2021;48:3630‐3637. 33993511 10.1002/mp.14945

[23] Vorbau R , Poludniowski G . Technical note: SpekPy web‐online x‐ray spectrum calculations using an interface to the SpekPy toolkit. J Appl Clin Med Phys. 2024;25:e14301. 38363037 10.1002/acm2.14301 PMC10929988

[24] Müller J , Schürer M , Neubert C , et al. Multi‐modality bedding platform for combined imaging and irradiation of mice. Biomed Phys Eng Express 2020;6:037003. 33438682 10.1088/2057-1976/ab79f1

[25] Brunner J , Langgartner L , Danhel H , et al. Dosimetric characteristics of 3D‐printed and epoxy‐based materials for particle therapy phantoms. Front Phys. 2024;12:1323788.

[26] Andreo P . Data for the dosimetry of low‐ and medium‐energy kV x rays. Phys Med Biol. 2019;64:205019. 31491771 10.1088/1361-6560/ab421d

[27] Bancheri J , Ketelhut S , Büermann L , Seuntjens J . Monte Carlo and water calorimetric determination of kilovoltage beam radiotherapy ionization chamber correction factors. Phys Med Biol. 2020;65:105001. 32208370 10.1088/1361-6560/ab82e7

[28] Damodar J , Odgers D , Pope D , Hill R . A study on the suitability of the PTW microDiamond detector for kilovoltage x‐ray beam dosimetry. Appl Radiat Isot. 2018;135:104‐109. 29413822 10.1016/j.apradiso.2018.01.025

[29] Niroomand‐Rad A , Chiu‐Tsao S‐T , Grams MP , et al. Report of AAPM task group 235 radiochromic film dosimetry: an update to TG‐55. Med Phys. 2020;47:5986‐6025. 32990328 10.1002/mp.14497

[30] Khachonkham S , Dreindl R , Heilemann G , et al. Characteristic of EBT‐XD and EBT3 radiochromic film dosimetry for photon and proton beams. Phys Med Biol. 2018;63:065007. 29474189 10.1088/1361-6560/aab1ee

[31] Resch AF , Heyes PD , Fuchs H , Bassler N , Georg D , Palmans H . Dose‐ rather than fluence‐averaged LET should be used as a single‐parameter descriptor of proton beam quality for radiochromic film dosimetry. Med Phys. 2020;47:2289‐2299. 32166764 10.1002/mp.14097 PMC7318138

[32] Nath R , Biggs PJ , Bova FJ , et al. AAPM code of practice for radiotherapy accelerators: report of AAPM radiation therapy task group no. 45. Med Phys. 1994;21:1093‐1121. 7968843 10.1118/1.597398

[33] Virtanen P , Gommers R , Oliphant TE , et al. SciPy 1.0: Fundamental Algorithms for Scientific Computing in Python. Nat Methods. 2020;17:261‐272. 32015543 10.1038/s41592-019-0686-2 PMC7056644

[34] Kataria T , Sharma K , Subramani V , Karrthick K , Bisht SS . Homogeneity index: an objective tool for assessment of conformal radiation treatments. J Med Phys. 2012;37:207‐213. 23293452 10.4103/0971-6203.103606 PMC3532749

[35] Lowekamp BC , Chen DT , Ibáñez L , Blezek D . The design of simpleITK. Front Neuroinform. 2013;7:45. 24416015 10.3389/fninf.2013.00045 PMC3874546

[36] Yaniv Z , Lowekamp BC , Johnson HJ , Beare R . SimpleITK image‐analysis notebooks: a collaborative environment for education and reproducible research. J Digit Imaging. 2018;31:290‐303. 29181613 10.1007/s10278-017-0037-8 PMC5959828

[37] Biggs S , Jennings M , Swerdloff S , et al. PyMedPhys: A community effort to develop an open, Python‐based standard library for medical physics applications. J Open Source Softw. 2022;7:4555.

[38] Chen Q , Molloy J , Izumi T , Sterpin E . Impact of backscatter material thickness on the depth dose of orthovoltage irradiators for radiobiology research. Phys Med Biol. 2019;64:055001. 30673636 10.1088/1361-6560/ab0120 PMC9202343

[39] Knäusl B , Langgartner L , Stock M , et al. Requirements for dose calculation on an active scanned proton beamline for small, shallow fields. Phys Med. 2023;113:102659. 37598612 10.1016/j.ejmp.2023.102659

[40] Lindsay PE , Granton PV , Gasparini A , et al. Multi‐institutional dosimetric and geometric commissioning of image‐guided small animal irradiators. Med Phys. 2014;41:031714. 24593718 10.1118/1.4866215

[41] Graves EE , Zhou H , Chatterjee R , et al. Design and evaluation of a variable aperture collimator for conformal radiotherapy of small animals using a microCT scanner. Med Phys. 2007;34:4359‐4367. 18072501 10.1118/1.2789498

[42] Wong J , Armour E , Kazanzides P , et al. High‐resolution, small animal radiation research platform with x‐ray tomographic guidance capabilities. Int J Radiat Oncol Biol Phys. 2008;71:1591‐1599. 18640502 10.1016/j.ijrobp.2008.04.025 PMC2605655

[43] Tryggestad E , Armour M , Iordachita I , Verhaegen F , Wong JW . A comprehensive system for dosimetric commissioning and Monte Carlo validation for the small animal radiation research platform. Phys Med Biol. 2009;54:5341‐5347. 19687532 10.1088/0031-9155/54/17/017 PMC3365538

[44] Kaveckyte V , Persson L , Malusek A , Benmakhlouf H , Alm Carlsson G , Carlsson Tedgren A . Investigation of a synthetic diamond detector response in kilovoltage photon beams. Med Phys. 2020;47:1268‐1279. 31880809 10.1002/mp.13988

[45] Price G , Biglin ER , Collins S , et al. An open source heterogeneous 3D printed mouse phantom utilising a novel bone representative thermoplastic. Phys Med Biol. 2020;65:10NT02. 10.1088/1361-6560/ab8078 PMC10606941 32182592

[46] Wegner M , Frenzel T , Krause D , Gargioni E . Development and characterization of modular mouse phantoms for end‐to‐end testing and training in radiobiology experiments. Phys Med Biol. 2023;68:085009. 10.1088/1361-6560/acc566 36930984

[47] Matinfar M , Ford E , Iordachita I , Wong J , Kazanzides P . Image‐guided small animal radiation research platform: calibration of treatment beam alignment. Phys Med Biol. 2009;54:891‐905. 19141881 10.1088/0031-9155/54/4/005 PMC2964666

[48] Clarkson R , Lindsay PE , Ansell S , et al. Characterization of image quality and image‐guidance performance of a preclinical microirradiator. Med Phys. 2011;38:845‐856. 21452722 10.1118/1.3533947 PMC3188651

[49] Kim H , Fabien J , Zheng Y , et al. Establishing a process of irradiating small animal brain using a CyberKnife and a microCT scanner. Med Phys. 2014;41:021715. 24506606 10.1118/1.4861713

